# Implementation strategies for integrating pre-exposure prophylaxis for HIV prevention and family planning services for adolescent girls and young women in Kenya: a qualitative study

**DOI:** 10.1186/s12913-022-07742-8

**Published:** 2022-03-30

**Authors:** Stephanie D. Roche, Gena Barnabee, Victor Omollo, Felix Mogaka, Josephine Odoyo, Elizabeth A. Bukusi, Jennifer F. Morton, Rachel Johnson, Connie Celum, Jared M. Baeten, Gabrielle O’Malley

**Affiliations:** 1 Department of Global Health, University of Washington, 325 Ninth Avenue, Seattle, WA 98104 USA; 2 Centre for Microbiology Research, Kenya Medical Research Institute, Nairobi, Kenya; 3 Department of Obstetrics and Gynecology, University of Washington, Seattle, USA; 4 Department of Medicine, University of Washington, Seattle, USA; 5 Department of Epidemiology, University of Washington, Seattle, USA; 6 Gilead Sciences, Foster City, USA

**Keywords:** Pre-exposure prophylaxis, Implementation science, Kenya, Delivery of health care, integrated, Family planning services, HIV infections

## Abstract

**Introduction:**

Across sub-Saharan Africa, ministries of health have proposed integrating pre-exposure prophylaxis (PrEP) for HIV prevention into family planning (FP) services to reach adolescent girls and young women (AGYW); however, evidence on effective implementation strategies is still limited. We conducted a qualitative study of integrated PrEP-FP service implementation at two FP clinics in Kisumu, Kenya.

**Methods:**

From June 2017 to May 2020, the Prevention Options for Women Evaluation Research (POWER) study enrolled 1000 sexually active, HIV-negative AGYW age 16 to 25. Actions taken to implement PrEP were captured prospectively in 214 monitoring and evaluation documents and 15 interviews with PrEP implementers. We analysed data using conventional and directed content analysis, with the latter informed by the Consolidated Framework for Implementation Research (CFIR) and the Expert Recommendations for Implementing Change (ERIC) compilation.

**Results:**

POWER deployed a variety of implementation strategies to train and educate stakeholders (e.g., having new providers shadow PrEP providers); develop stakeholder interrelationships (e.g., organizing support teams with protected time to reflect on implementation progress and make refinements); provide technical assistance; and change physical infrastructure and workflow. Although these strategies reportedly influenced contextual factors across four of the five CFIR domains, they primarily interacted with contextual factors relevant to inner setting, especially implementation climate and readiness for implementation. Overall, implementing PrEP proved easier and less labor-intensive at a private, youth-friendly clinic than a public FP clinic, largely because the baseline structural characteristics (e.g., space, workflow) and organizational mission of the former were more conducive to offering AGYW-centered care. Nevertheless, adoption of PrEP delivery among non-study staff at both sites was low, likely due to the widespread perception that PrEP was not within their scope of work.

**Conclusions:**

Some FP clinics may be “lower-hanging fruit” than others for PrEP implementation. Approaching PrEP implementation as a behavioral intervention for FP providers may help ensure that providers have the requisite capability, opportunity, and motivation to adopt the clinical innovation. In particular, PrEP implementers should assess the need for implementation strategies that support providers’ clinical decision-making, establish worker expectations and accountability, and address workload constraints.

**Trial registration:**

Clinical Trial Number: NCT03490058.

**Supplementary Information:**

The online version contains supplementary material available at 10.1186/s12913-022-07742-8.

## Introduction

In eastern and southern Africa, adolescent girls and young women (AGYW) bear a disproportionate burden of the HIV epidemic. In 2019, AGYW age 15 to 24 accounted for 26% of new infections and were 2.5 times more likely to acquire HIV than their male counterparts [1]. Per WHO recomendations [2], many countries in the region have designated AGYW as a priority population for daily oral tenofovir-based pre-exposure prophylaxis (PrEP) for HIV prevention [3]. Since 2014, substantial investments have been made to expand PrEP services to AGYW in this region [4].

Research about PrEP delivery to this population has focused heavily on (1) understanding end-users and (2) tailoring delivery approaches to meet their care needs and preferences. Studies in this first domain have measured PrEP use (e.g., uptake, adherence, continuation) and its predictors [5–7] and engaged AGYW to understand their PrEP decision-making [6, 8–10]. Qualitative investigations have identified a variety of barriers to AGYW PrEP use, such as low HIV risk perception [11–13]; high stigma for being sexually active [14]; lack of financial independence [15]; and low social support for PrEP use [13, 16–18]. These findings have motivated the second major area of research, centered on developing and testing delivery models and interventions to support AGYW in PrEP use. These models vary not only by setting of PrEP provision (e.g., community safe spaces, mobile clinics) and types of providers involved, but also by services offered alongside PrEP and use of delivery innovations, such as digital pillboxes with SMS reminders, to support adherence [19].

Although our understanding of the “demand-side” of the PrEP delivery equation has grown steadily over the past decade, our understanding of the “supply-side” has not kept pace. In particular, our knowledge about supply-side implementation strategies—actions for enhancing the adoption, implementation, and sustainability [20] of PrEP delivery—is still nascent. Beyond where PrEP is delivered and by whom, most PrEP initiatives do not provide granular detail about implementation strategies used or how these strategies affected, and were affected by, contextual factors relevant to implementation, such as organizational culture. Yet, this level of detail is fundamental for helping PrEP implementers understand the “how” of PrEP implementation and for informing their decisions about where to scale. Due to the broad scope of our research objective and the lack of evidence on contextual factors and implementation strategies relevant to PrEP delivery in our study setting, we opted to frame our research around two implementation science frameworks, selected for their breadth and potential relevance to multiple levels of influence on implementation (e.g., at the individual, organizational, and policy levels). The Consolidated Framework for Implementation Research (CFIR) is a meta-theoretical framework of 39 constructs (or “determinants”) hypothesized to predict, moderate, or drive implementation outcomes [21, 22]. To optimize the CFIR for use in low- and middle-income settings, Means et al. incorporated an additional 11 constructs [23]. The Expert Recommendations for Implementing Change (ERIC) compilation [24, 25] is a compendium of 73 discrete implementation strategies that can be used prospectively to support identification of strategies to use or retrospectively to support comprehensive reporting of strategies used [26]. Perry et al. subsequently revised the ERIC compilation and introduced three additional strategies [27].

We utilized both frameworks to evaluate the implementation of integrated PrEP-family planning (FP) services at two FP clinics in Kenya. From June 2017 to May 2020, the Prevention Options for Women Evaluation Research (POWER) study enrolled 2469 sexually active, HIV-negative AGYW age 16 to 25 in Kisumu, Kenya (*n* = 1000) and Johannesburg and Cape Town, South Africa (*n* = 1469). Because the Kenya Ministry of Health (MOH) is interested in scaling PrEP to FP clinics [28], this sub-study focuses on POWER’s two FP clinic sites. Our objective was to identify the supply-side implementation strategies employed by POWER, map these to contextual determinants, and understand the potential implications for scale-up.

## Methods

### Study setting

POWER’s two Kenya sites were located in Kisumu County, where HIV incidence is 6.3 per 1000 population—3.5 times the national average [29]. Site A was an FP clinic housed within the Maternal and Child Health Department of a public referral hospital. Site B was a youth-friendly FP clinic within a private, NGO-run health facility. At both sites, POWER introduced paid project staff to help design clinic flows that site staff anticipated would mitigate potential barriers to PrEP implementation and demonstrate PrEP delivery to site staff.

### Data collection

This study uses two data sources. From April 2017 to June 2020, we collected routine reports and notes from meetings and calls among Kisumu POWER staff to discuss program monitoring and evaluation (M & E). These M & E documents were generated in real time by various individuals, commonly the Seattle-based research manager and Kenya-based study coordinators.

From October to December 2019, we conducted 15 key informant interviews (KIIs). Our sampling frame included both facility staff and POWER staff (employed by the study) involved in PrEP service delivery and/or program implementation. Eligible individuals were age 18 or above and self-reported comfort communicating in English. We used purposive sampling to recruit participants of different roles and primary employers. The Kenyan study coordinator contacted eligible individuals, described the study’s purpose, and informed them that they would be contacted by an external research assistant (RA) about participating in a confidential interview. The RA (author SDR) was an American, female PhD student with doctoral-level training in qualitative research and no prior relationship with interviewees. She conducted all interviews one-on-one, in English, in a private room or via phone call, using a semi-structured interview guide (Appendix A of Additional file 1) informed by the CFIR framework [21, 23]. The guide solicited information on participants’ role in PrEP implementation; perceived advantages and disadvantages of the delivery model; strategies used to deliver PrEP; and recommendations for scale-up. Interviews typically lasted 85 min (interquartile range: 67–90 min), were audio recorded, and transcribed verbatim.

### Data analysis

We analyzed data using a combination of conventional content analysis and directed content analysis [30], with the latter informed by Perry et al.’s modified ERIC compilation [25, 27] and Means et al.’s modified CFIR framework. After repeated readings of the data [31], two experienced qualitative analysts (authors SDR and GB) developed an initial codebook of deductive codes (relevant CFIR determinants and ERIC strategies) and inductive codes. They independently applied the codebook to subsets of the data, compared their coding for consistency, and refined the codebook, as needed. All documents were coded in ATLAS.ti v.8 (Scientific Software Development GmbH, Berlin, Germany) by SDR and reviewed by GB. Comparisons between Sites A and B were descriptive and based solely on the study’s two aforementioned data sources. Additional details about our methodology are available in eTable 1 of Additional file 1.

### Ethics

The institutional review boards of the University of Washington and the Kenya Medical Research Institute approved this study. Written informed consent was obtained from interviewees, who were compensated 500 Kenyan shillings (about USD $4.50) for participating. M & E activities were determined to be exempt.

## Results

We collected 214 M & E documents (Table 1) and conducted 15 interviews (Table 2). Our interviewee sample was predominantly female (9/15, 60%) and employed by the POWER study (10/15, 67%). All individuals who were invited agreed to participate in an interview. We identified four major themes related to (1) the learning environment and provider support; (2) the physical environment and clinic flow; (3) providers’ perceived scope of work; and (4) workload. Within each theme explanation, we reference key differences between Sites A and B at study baseline (Table 3), some of which drove implementation strategy selection, the strategies implemented, and the CFIR determinants these strategies influenced (the latter two of which are also summarized in Table 4).

**Table 1:** Monitoring and evaluation (M & E) documents collected and analyzed

M & E activity			Documentation (main author)	No. (%) documents (***N*** = 214)
Frequency & type	Participants	Aim		
Biweekly meeting	Site study coordinator, study staff involved in direct service provision	To review biweekly enrollment numbers and service delivery-related activities undertaken since last meeting (e.g., PrEP information sessions held for non-POWER staff to raise awareness about PrEP/its availability at the site).	Meeting notes (project coordinator and site study coordinator)	76 (36)
Monthly call	Full POWER research team^a^	To discuss the research study’s progress (e.g., enrollment numbers) and preliminary findings; to obtain consensus about analytic questions (e.g., different ways to calculate PrEP continuation and the pros/cons of each); to discuss changes in Kenya’s/South Africa’s PrEP delivery landscape (e.g., to national PrEP guidelines); and to conduct and provide feedback on dry-runs of study presentations.	Call notes (various authors)^a^	53 (25)
Monthly call	Project coordinator, site study coordinator, study staff involved in direct service provision	To discuss study operations (e.g., stock levels for study commodities; shipping permits for sending dried blood spot samples to the lab for analysis; ensuring study is in compliance with regulatory agencies).	Call notes (project coordinator)	33 (15)
Monthly call	Site study coordinators, project coordinator, research assistants from Kenya & South Africa sites	To share experiences, challenges, and lessons learned about implementing integrated delivery of PrEP-FP services.	Call notes (site study coordinators)	27 (13)
Quarterly meeting	Study site coordinators, study staff involved in direct service provision	To discuss quarterly enrollment data and high-level challenges to implementation (e.g., nursing strikes) and agree upon next steps.	Meeting notes (site study coordinators)	16 (7)
Annual meeting	Full POWER research team^a^, other key stakeholders^b^	To present study findings to date.	Slide decks (various authors)^a^	9 (4)

^a^Includes PIs, project manager, project coordinator, site study coordinators, study staff involved in direct service provision, research assistants, and other co-investigators

^b^e.g., representatives from the Ministry of Health and local Community Advisory Boards

**Table 2:** Demographic characteristics of interviewees (*N* = 15)

Characteristic	Value
POWER as primary employer – no. (%)	10 (67)
Primary occupational role^a^ – no. (%)	
Healthcare provider	10 (67)
Clinician (nurse or clinical officer)	6 (60)
HTS counsellor	3 (30)
Other counsellor	1 (10)
Other key informant^b^	5 (33)
Primary site affiliation – no. (%)	
Site A	6 (40)
Site B	6 (40)
Both	3 (20)

^a^Based on participant’s primary role vis-à-vis PrEP delivery and the POWER study. For example, a participant who is a doctor by profession but whose primary role in POWER was a study coordinator is counted as “other key informant”

^b^Held administrative roles within the POWER study or at a study site

**Table 3:** Key differences between Site A and Site B at study baseline

Characteristic	Site A	Site B
Sector	Public	Private, non-profit
Facility type	Regional teaching and referral hospital	Stand-alone facility
Governing body	MOH	Executive board
Services offered at facility	Wide range of primary, secondary, and tertiary care (e.g., diabetes screening, pediatric oncology, intensive care)	Primary and secondary care focused on reproductive, maternal, neonatal, child and adolescent health services
PrEP delivery history	Prior to and during POWER study, PrEP also offered at hospital’s HIV clinic and in modular booths at hospital entrance	Facility did not offer PrEP prior to POWER study
Where POWER study delivered PrEP -Description	Outpatient/MCH department −13 consultation rooms (2 specifically for FP), 6 waiting bays, 2 HTS points, 1 lab, 1 pharmacy	Youth-friendly clinic (YFC) −2 consultation rooms and 1 waiting bay. Lab and pharmacy in separate clinic area serving entire clinic.
FP client volume	~ 50 FP clients per day	~ 4 FP clients per day
Pre-study FP service delivery configuration	Clients check in at registration desk, then move to different service delivery points for HTS (mandatory), FP services, lab exams (if needed), and pharmacy services (if needed).	Clients go directly to YFC and receive FP services and, if needed, HTS in the same room by same provider or with providers coming to them. Clients move for lab exam and pharmacy services, as needed.
Baseline plan for PrEP integration	Train FP providers to deliver integrated PrEP-FP services (e.g., counsel clients about both at the same time), with clients continuing to receive HTS, lab, and pharmacy services from their respective service delivery points.	PrEP added to existing bundle of services offered to AGYW clients that included HIV testing and counseling, FP counseling, and cervical cancer screening.

**Table 4:** Implementation strategies used and determinants influenced, according to participants

ERIC strategy number	ERIC strategy name	CFIR determinant(s) influenced	Description	Illustrative quotes
19	Conduct ongoing training	+ Knowledge and beliefs about intervention	POWER staff periodically held trainings to educate site staff about PrEP and encourage them to refer clients for PrEP services.	*At first, I was like, “Why are they giving people ARVs before they are sick? They will form resistance and that will be an issue.” We [FP nurses] did not understand PrEP until they [POWER staff] gave us the CME [continuing medical education]. Then I learned that this thing actually protects people from getting HIV. … Once I learned what it was, I really embraced it.* (Nurse, not employed by POWER, IDI 4)
54	Provide local technical assistance	+ Access to knowledge and information	POWER study coordinator was available on-demand to answer providers’ questions and assist with complex cases.	*[If I had questions,] I normally turned to the study coordinator. All my questions have always been answered, especially the technical ones, because we need an expert in the medical field to assist at times.* (Counselor, employed by POWER, IDI 2)
48^a^ 5	Organize implementation teams and team meetings Audit and provide feedback	+ Reflecting and evaluating + Goals and feedback	POWER staff held weekly meetings to review M & E reports, discuss challenges, and devise improvement plans to reach their goal of enrolling 1000 AGYW.	*Through the week, we come with the challenges, the successes. We are getting the information from clients when we are making follow-ups. So in the [weekly] POWER meeting, we come and weigh, “How should we do this to make a change in this?” [If the M & E report shows high lost-to-follow-up rates, we discuss,] “How can we make these clients who are missing to come back for PrEP?”* (HTS provider, employed by POWER, IDI 7)
20	Create a learning collaborative	+ Learning climate +Cosmopolitanism	POWER staff from Kenya and South Africa study sites shared implementation challenges experienced and lessons learned during monthly calls.	*[A staff member from the Kenyan sites] reported that, for the past 2 months, they’ve had a lot of follow-up visits. … [A South African colleague] asked how the clients they enrolled during recent outreach activities are different from the ones they had trouble retaining in the past. [The Kenyan colleague] said they are doing more intense [HIV risk] screening and counseling to try to make sure the people [they enroll] are really interested in PrEP. They’ve found that those who come [to the clinic to initiate PrEP] later [*i.e.*, in the days after the outreach event] tend to be more dedicated than the ones who they got on the day of the event. … So now the girls who enroll are doing so because they want to enroll, not because they feel like they need to.* (Call notes, December 2018)
74^b^ 11	Assess and redesign workflow Change physical structure and equipment	+ Patient needs and resources -Perceived sustainability^c^ (Site A only)	POWER staff worked with sites to reorganize service delivery to meet AGYW care preferences (e.g., privacy, short service times), though some interviewees expressed concern about sustainability.	*When we saw that working within the FP space was not going well, [the head of Site A’s outpatient department] gave us the other room [*i.e.*, a separate room for PrEP delivery] for privacy. … Queuing at the pharmacy was also a challenge to our participants because those who come … for ARVs would take at that [same] pharmacy. So stigma was there. [Clients worried,] “They will see as if I’m also taking ARVs.” So we decided we’d let the nurse or the clinician prescribe and dispense the medication [from the private room]. Clients preferred that.* (Other key informant, employed by POWER, IDI 17) *Transitioning [*i.e.*, ending POWER’s involvement in PrEP delivery] may be a challenge because the queueing we were trying to avoid [clients from having to do], now it will force them [to queue].* (Counselor, employed by POWER, IDI 7)
40	Involve executive boards	+ Relative priority	At Site B, leaders had POWER staff report out PrEP implementation progress at weekly all-staff meetings.	*[Site B leaders] are like 100% [in favor of PrEP]. They even ask for feedback at the weekly [all-staff] meeting: ‘How is it [PrEP delivery] going?’ And sometimes when we reach our [enrollment] target, they tell us [POWER staff] to stand up and the whole team appreciates [*i.e.*, applauds us].* (Counselor, employed by POWER, IDI 12)
60	Shadow other experts	+ Knowledge and beliefs about intervention - Relative priority -Perceived sustainability^c^ (Site A only)	The study hired nurses and HTS providers to introduce PrEP delivery at study sites so site providers could observe how to deliver PrEP (e.g., how to counsel AGYW about PrEP). This infusion of human resources, however, may have lowered some providers’ sense of responsibility towards PrEP delivery. For some interviewees, it also raised some concerns about sustainability.	*[POWER] brought in some other staffs for them [providers at Site B] to shadow … to have a feel of it [PrEP delivery] and understand that there is not much work [involved in delivering PrEP]. … And some [providers] bought [into] the idea. … So bringing in other people to overshadow each one of them, I think those played a role [in facilitating PrEP implementation].* (Counselor, employed by POWER, IDI 12) *As much as we had our own responsibilities, I just got to a point and told myself that having knowledge will not hurt you in any way. … The POWER staff were mentors. They guided us [on] how do you enroll? How do you counsel? What do you tell these girls? So that is how I got to learn [about PrEP delivery] and started helping out here. But for the other staff, it was just an added responsibility. Already they had their jobs, the things they had to do. … They really associated PrEP with POWER.* (Clinician, not employed by POWER, IDI 1) *The POWER study was able to offer timely services because they had more healthcare workers. When that is translated to a normal MOH facility without such support, that may negatively affect it because people [clients] will have long waiting hours and may start to prioritize other things [over PrEP].* (Other key informant, not employed by POWER, IDI 16)

^a^Strategy name modified by Perry et al.

^b^Strategy added to ERIC framework by Perry et al.

^c^Construct added to CFIR framework by Means et al.

### Theme 1, learning environment & provider support

At both sites, providers benefitted from a mix of support services to enable PrEP delivery, including urgent, on-demand clinical decision-making support and routine, cross-site meetings to strategize about ongoing implementation.

According to interviewees, frequent PrEP trainings (**ERIC strategy**: ***Conduct ongoing training***) held by POWER staff helped dispel some providers’ initial safety concerns about offering an ARV-based product to healthy individuals (**CFIR**: ***Knowledge and Beliefs about the Intervention***). However, to successfully deliver PrEP, providers needed more than just accurate knowledge and positive beliefs about PrEP’s safety and efficacy. Interviewees described sometimes encountering cases that required additional PrEP expertise, most commonly for determining client eligibility to initiate and/or continue PrEP. In these situations, they turned to the study coordinator, a trained clinician who provided local technical assistance (**ERIC strategy:** ***Provide local technical assistance*; CFIR**: ***Access to Knowledge and Information***) in the form of on-demand clinical decision-making support:

*When providers … had a client with a positive Hepatitis B test, or a pregnant lady that had questions that they could not answer, or a client who had side effects and they wanted my input, I would go and see the client.* (POWER study coordinator)

M & E documents indicate that the study coordinator also coached providers in their PrEP delivery approach, advising them, for example, to give clients ample autonomy in their decision-making:

*One nurse declined to issue [PrEP] because she [the client] did not have somewhere to store PrEP in her house and did not intend to tell her husband. [The study coordinator] advised that no one should be denied PrEP because of lack of somewhere to store it … so participants should just be given PrEP if they need it, and they will find a way of dealing with their issues at home.* (Meeting notes, November 2017)

Providers also reported that having protected time to reflect on progress facilitated PrEP implementation (**CFIR**: ***Reflecting and Evaluating***). POWER providers had a weekly standing meeting to review M & E reports (**ERIC strategies**: ***Audit and provide feedback; organize implementation teams and team meetings***) and discuss how to improve PrEP delivery (**CFIR**: ***Goals and Feedback****)*:

*There’s constant feedback [on implementation]. There’s a weekly report showing us how many [clients] we enrolled, how many are continuing, how many discontinued or restarted. We look at this overview of how our work is going and usually find areas to improve on. Like if the retention has gone down, we see on how to restructure [delivery] and what can work best.* (Male POWER staff, KII 6)

M & E documents further detail how provider capability to implement PrEP was enhanced by shared learning between the Kenya and South Africa sites. During monthly calls, members of this learning collaborative shared their implementation experiences, including successes and challenges (**ERIC strategy**: ***Create a learning collaborative*; CFIR**: ***Learning Climate***). Common topics included how to reach potential PrEP clients and support clients’ continued PrEP use. Tactics that worked well at one site were often subsequently tried at others (**CFIR**: ***Cosmopolitanism***). Overall, many interviewees felt that successful implementation of PrEP in other FP clinics would require ensuring providers have access to PrEP experts for clinical decision-making support and protected time to discuss and troubleshoot challenges, especially in the beginning months of implementation.

### Theme 2, physical environment & clinic flow

Site B’s designated youth-friendly space and clinic flow, which required less room-to-room movement for clients, made implementing PrEP delivery to AGYW easier compared to Site A, which required a series of changes to its physical environment to create an acceptable clinic experience for AGYW.

Overall, integrating PrEP into routine FP services required fewer implementation strategies at Site B than Site A. Characteristics that appeared to make Site B better positioned at the outset included its designated private space for serving youth and its existing clinic flow, which entailed less room-to-room movement for clients. As such, to add PrEP services, Site B did not need to reconfigure its clinic space or make major changes to how clients and providers move through service delivery.

Initially, Site A offered PrEP like any other outpatient service, with clients receiving HIV testing services (HTS) at HTS points, PrEP counseling and clinical review in consultation rooms, and prescription dispensing at the pharmacy. Providers soon found, however, that this delivery configuration was not acceptable to AGYW who were not keen on queueing at each service delivery point and who did not want to discuss their sexual activity in crowded FP consultation rooms (**CFIR**: ***Patient Needs and Resources***). Site A, therefore, adapted its workflow and space configuration (**ERIC strategies**: ***Change physical structure and equipment****;* ***assess and redesign workflow***), acquiring a separate, private room for PrEP clients:

*We realized that the FP rooms are too small because there are a lot of interns [nursing students] in there with the nurse. There was not enough privacy. So the in-charge gave us another room.* (Call notes, November 2017)

Site A also began fast-tracking clients to the front of HTS queues and implemented in-room PrEP dispensing so clients could skip the pharmacy. A few interviewees, however, expressed uncertainty as to whether Site A would, in the long-term, be able to maintain this new delivery configuration without hiring additional staff (**CFIR**: ***Perceived Sustainability***). Notably, when asked what they thought would be essential to scaling PrEP delivery to other FP clinics, several interviewees highlighted the importance of streamlined service delivery:

*[It will be important] to look at the setting and how well it will suit PrEP delivery in terms of privacy and waiting time. Because those are the little things that contribute a lot, especially the waiting time. If a client waits for long, then the client will disappear, even if they really are at risk [of HIV].* (Male POWER staff, KII 6)

### Theme 3, perceived scope of work

At both sites, getting providers to fully and consistently engage in PrEP delivery was challenging, in part, because most viewed PrEP delivery as falling outside their scope of work. Bringing in additional staff to demonstrate how to deliver PrEP to AGYW may have inadvertently lowered providers’ sense of responsibility toward PrEP delivery.

Throughout the study, engagement of non-POWER staff in PrEP delivery was low. The study’s original intent was to have POWER staff demonstrate PrEP delivery to providers at each site (**ERIC strategy**: ***Shadow other experts***) and for these providers to assume an increasingly larger role in PrEP delivery over time; however, by the study’s end, only a few providers at Site B took to PrEP delivery and attended to PrEP clients on their own. More commonly, staff referred clients to POWER staff who, ultimately, conducted the bulk of PrEP education, counseling, and clinical review at both sites:

*We [POWER staff] are not supposed to deliver PrEP ourselves. We are supposed to bring these guys [site staff] on board to deliver PrEP. … [But] getting staff to embrace this PrEP thing, we have been struggling. … Some feel like it is an added work. … If the client tells them, “I’ve heard about this thing called PrEP—" they send the client over to us [instead of attending to the client themselves].* (Female POWER staff, KII 8)

Providers at Site B generally agreed with their clinic’s decision to add PrEP services, while providers at Site A did not. Whether providers perceived PrEP delivery to fall within their scope of work appeared to be influenced by five key factors, described below. These factors, which were identified inductively from the data, do not fit perfectly within the CFIR framework; however, where applicable, we indicate related CFIR determinants.

#### *Alignment with organizational mission* (**Related CFIR determinant**: ***Compatibility***)

Interviewees reported that providing PrEP to AGYW aligned with Site B’s organizational mission to holistically support adolescent sexual and reproductive health (SRH):

*[Site B’s] core goal is serving young women. … ensuring their safety and well-being. So adding PrEP to [Site B] was viewed as great for girls and women.* (Female POWER staff, KII 1)

The organizational mission of the MCH department of Site A, by contrast, was broadly focused on providing MCH and FP services, with no specific emphasis on HIV prevention or adolescent health.

#### *Experience with integrated service delivery* (**Related CFIR determinant**: ***Knowledge and Beliefs about the Intervention***)

Some interviewees hypothesized that Site B providers were generally more open to adding PrEP services because providers were already accustomed to offering a bundle of SRH services whose composition shifts from time to time:

*[At Site B], we offer integrated services … We always promote our services as a package to promote good health. …. And now that package includes PrEP.* (Male non-POWER staff, KII 9)

At Site A, however, integrated service delivery was not the norm. As such, PrEP delivery represented, in the eyes of some Site A providers, an entirely new set of responsibilities:

*Some health personnel [at Site A] view it as extra work because, for them, PrEP is another whole package. You need to confirm HIV status. You need to assess HIV risk. You need to counsel. So some of them feel like it is a lot of work.* (Female POWER staff, KII 3)

#### Leadership engagement

Although POWER staff engaged leadership (**ERIC strategy**: ***Involve executive boards*; CFIR**: ***Leadership Engagement***) at both sites, Site B leaders reportedly took a more active role in promoting PrEP delivery. For example, Site B leaders had monthly PrEP enrollment numbers publicly displayed and reported at weekly all-staff meetings:

*[At Site B] the director is very supportive [of PrEP delivery]. We have all-staff meetings every week, and she wants to know what’s going on, how we’re faring. … And she tells everyone, “We need to work together on this for the betterment of AGYW.” So others hear it right from the top.* (Female POWER staff, KII 8)

According to interviewees, Site A leaders also supported PrEP delivery. For example, they welcomed POWER staff to hold trainings and information sessions and, early on, allocated a private room for PrEP delivery; however, their support for PrEP delivery was generally more subtle and potentially less visible to other staff.

#### Youth-friendly service delivery skills

Lastly, Site A and B providers differed in that the latter were generally more experienced with delivering youth-friendly SRH services. As such, they reportedly had fewer moral reservations about providing PrEP to AGYW than Site A providers, many of whom had initial concerns about promoting “promiscuity.” POWER staff reported assuaging some of these concerns over time, often through one-on-one conversations in which they appealed to providers’ sense of empathy and professional duty to protect AGYW from HIV.

#### Delineation of worker responsibilities

Although support for adding PrEP services for AGYW was higher among Site B providers, such endorsement did not directly translate to provider adoption of PrEP delivery. Interviewees attributed low provider engagement in PrEP delivery to a widespread perception among providers at both sites that PrEP was not within their scope of work, or at least not while the POWER study was ongoing. As outsiders, POWER staff lacked the authority to formally update providers’ job descriptions to include PrEP responsibilities or hold providers accountable if they did not engage in PrEP delivery. Most site staff, therefore, opted to prioritize delivering services that had historically been included in their scope of work (**Related CFIR determinant**: ***Relative Priority***). When asked about scaling PrEP delivery to other FP clinics, some interviewees highlighted the need for management to clearly communicate that PrEP delivery is part of their job:

*There should be adjustments in that [PrEP] responsibilities should be given to the existing staffs so that they add onto their role. [Staff should be told,] “Despite your doing this, this [PrEP delivery] is also your department.” … They [managers] have to talk to them and sensitize them on what they want [providers] to do so that they know, “This is what is supposed to be done.”* (Female non-POWER staff, KII 5)

The presence of POWER staff may have inadvertently reinforced providers’ perception that PrEP delivery was not within their scope of work. Because POWER staff were being paid to implement PrEP during the study, site providers reportedly viewed POWER staff’s requests to engage in PrEP delivery as akin to dumping their responsibilities onto them:

*Some providers would say, “No, we can’t [deliver PrEP]. We are not paid by the POWER team, so we can’t do their work.”* (Female POWER staff, KII 7)

Lastly, providers’ perception of PrEP delivery as “unpaid, extra work” may also have been fueled by a precedent set by other research studies that paid site staff to assist with service delivery. Indeed, throughout the first year of the study, POWER staff had to repeatedly decline requests from site staff for additional pay:

*One challenge we’re experiencing is that one of the HTS providers [at Site A] is always asking to be reimbursed whenever he refers a participant for PrEP. We’ll make him aware that there is no reimbursement and whatever he does is part of his work.* (Call notes, February 2018)

### Theme 4. Workload

Even providers who are willing to deliver PrEP to AGYW may be thwarted by existing high workloads. Overcoming this barrier may necessitate hiring additional staff and/or finding ways to lighten provider workload.

POWER staff reported that some portion of providers’ low engagement in PrEP delivery was likely due to high workloads. Throughout the study, workload-related service delivery disruptions and delays were common at both sites:

*At the end of the month, clients struggle to be served at [Site B’s HTS point]. HTS providers are busy doing reports. … Currently, PrEP clients coming for follow-up have to go to another [department’s] testing point.* (Meeting notes, May 2019)

Most interviewees, therefore, anticipated that workload would be a barrier if PrEP were introduced to other FP clinics, especially public ones:

*Getting them [FP providers at Site A] involved [in PrEP delivery] was tough. And it’s tough up to now. So if you want to transition PrEP [delivery] to the government, it will still be difficult for them because of the workload. Because when you put it [PrEP] together with family planning, … it actually needs understanding. You have to talk to this lady. They have to ask questions, and you have 70 other women to attend to. It is not easy.* (Female POWER staff, KII 18)

When asked what it would take to successfully implement PrEP in other FP clinics, nearly all interviewees emphasized the need for sufficient human resources and anticipated these would have to come from outside:

*[FP clinics adding PrEP services] will need to employ more staff because, at the end of the day, the quality of healthcare is determined by the number of people [delivering and receiving services]. You see, nurses are human beings; lab technicians are human beings. They can only do so much. … But if there are enough [staff] compared to the patient ratio, at least providers wouldn’t have the issue of straining or burnout.* (Female POWER staff, KII 3)

Acknowledging that hiring staff may not be feasible, a few participants recommended that PrEP implementers explore other ways to lighten providers’ workload:

*Human resources can be a constraint. … [So it will be important to look at,] “How many [clients] do you have? How many people can they see in what sort of through-put time?... We may not be able to hire staff or change the remuneration, but what are some of the other things that help people be able to do a good job? And it comes down to things like good working environment. … How do we protect that time for a healthcare worker [to attend to] a woman who walks in and requires PrEP and is going to take an hour, when they could have seen 10 other people [in that time]. So there is that genuine pressure around time that needs to be addressed.* (Female POWER staff, KII 13)

## Discussion

Across sub-Saharan Africa (SSA), PrEP implementers eager to reach AGYW are in the early stages of integrating PrEP into routine FP services. Our study adds to the literature on integrated PrEP-FP service delivery by linking two frameworks—the ERIC compilation [25] and the CFIR [21]—to describe how certain implementation strategies affected, and were affected by, a range of determinants at two FP clinics in Kenya and how this interplay shaped the overall implementation process. Though specific to Kenya, our findings may help inform other countries’ approaches to implementation, particularly if the goal is to scale to both public and private facilities.

Our study found that new PrEP providers benefitted from clinical decision-making support, suggesting that training alone may be insufficient to enable providers to routinely and independently deliver PrEP. Light touch support early on may help new PrEP providers “get their feet wet” and create the right learning environment for them to quickly gain confidence in their ability to deliver PrEP. PrEP implementers could, for example, avail subnational-level PrEP technical advisors in person or remotely. Such clinician support services have proven successful in other settings, such as the California-based National Clinician Consultation Center, which, since 1993, has provided free and confidential phone consultations to providers on HIV testing and prevention, treatment, co-infections, post-exposure prophylaxis, and PrEP [32].

Whereas the current literature on integrated PrEP-FP delivery often refers to “FP clinics” as a uniform delivery setting, our study revealed key points of heterogeneity between two FP clinics that influenced implementation (e.g., organizational mission, clinic flow). Our finding that the youth-friendly clinic was potentially “low-hanging fruit” for this HIV prevention innovation is consistent with other projects and programs in Kenya [6], South Africa [33, 34], and Malawi [35], which similarly reported that providers at these venues were generally open to delivering PrEP and that AGYW like obtaining services in these settings. The implications of our findings for scale-up are even clearer when placed within the context of a typology of health facilities recently developed by Dunbar et al. based on their assessment of 20 facilities in Kenya and Zimbabwe [36]. This typology places facilities on a “youth-friendly and HIV/SRH integration continuum,” ranging from 1 to 5, and presumably draws on the WHO’s definition of adolescent-friendly health services as ones delivered by healthcare providers who are “non-judgmental and considerate in their dealings with adolescents” and in facilities that “are equipped to provide adolescents with the health services they need and are appealing and ‘friendly’ to adolescents.” [37], ^(p.6)^ Using this continuum, we see that POWER Site A was, at study baseline, a “Level 1” facility in that it did not have any special accommodations for providing youth-friendly services and, if clients needed more than one type of service, they were referred internally, without being escorted to, or fast-tracked at, the next service delivery point. By contrast, POWER Site B was, at baseline, a “Level 4” facility because, there, providers trained in youth-friendly service delivery offered fully integrated services in private, “youth only” spaces. Through this lens, we see that several of the implementation strategies employed at POWER Site A were, in effect, an attempt to make this “Level 1” facility more like a “Level 4” facility. Ultimately, POWER staff managed to streamline PrEP delivery at Site A and convinced providers to assume a more youth-friendly attitude toward AGYW PrEP use. The effort to achieve this, however, was substantial, and the sustainability of this new service delivery configuration without additional staffing remains unclear. As countries scale up integrated PrEP-FP services, it will be important to consider the potentially heavier “lift” of implementing at facilities lower down on this continuum and to plan accordingly. More research is needed to better understand how much additional effort (and cost) it takes to successfully implement at lower- vs. higher-level facilities.

Our finding that POWER staff struggled to get providers at the clinic to assume PrEP delivery responsibilities echoes the early experiences of other PrEP projects (including ones that did not add staff) [38, 39] and serves as a reminder that implementing integrated PrEP-FP services requires FP providers to view PrEP as part of SRH services and, thus, within their scope of work. The low adoption of PrEP delivery practices among non-POWER providers indicates that the bundle of implementation strategies employed by POWER did not sufficiently address provider motivation. Here, Michie et al.’s concept of “intervention function” is useful. Using behavioral science theory, the authors identified nine “intervention functions” or means by which an intervention can change behavior. In reviewing the implementation strategies POWER employed, we see that most had education, persuasion, or modeling functions. Few, however, had an incentivization function (creating expectation of reward), coercion function (creating expectation of punishment or cost), or restriction function (using rules to increase engagement in PrEP delivery or decrease engagement in competing behaviors). The common provider perception that PrEP delivery was not within their scope of work suggests that getting providers to adopt this behavior may require leaders/managers to employ additional strategies, such as “mandate change” (ERIC strategy 44) to formally revise providers’ job responsibilities, “audit and provide feedback” (ERIC strategy 5) to hold them accountable, and “alter incentive/allowance structures” (ERIC strategy 2) as part of broader carrot-and-stick approaches to motivate behavior change. Furthermore, our study suggests that bringing in outside staff to assist with PrEP implementation should be used with caution (e.g., with a timeline for transition of responsibilities).

Evidence from other PrEP implementation projects [40, 41] lend credit to POWER staff’s concern that high provider workloads could derail implementation. For example, “increased workload and documentation burden amid healthcare workforce shortages” emerged as a key challenge in the PrEP Implementation for Young Women and Adolescents (PrIYA) study, which, from 2017 to 2018, integrated PrEP into 16 MCH/FP facilities in Kisumu, Kenya [42]. Drawing again from Michie et al.’s theory of behavior change, this finding suggests that, before asking providers to take on the additional work of PrEP delivery, implementers should ensure that the delivery environment affords providers sufficient “physical opportunity” [43], ^(p.63)^ (e.g., time, access to necessary resources) to adopt the desired behavior. For example, implementers could conduct time-and-motion studies [44, 45] to measure how much, if any, available time providers have for PrEP delivery. If providers are at (or very close to) maximum capacity and clinics are unable to hire additional staff, then implementation is unlikely to succeed unless other clinical responsibilities are removed from providers’ workloads and/or inefficiencies eliminated from provider workflow. A variety of strategies, including some already in use in SSA, may be pursued to this end, including shifting PrEP tasks to lower-level cadres (e.g., peer educators) [46], modifying clinical practices (e.g., adopting multi-month scripting to reduce client volume) [46–48], and using client-facing interventions, such as HIV self-testing [49] and decision-support tools [50], to expedite the clinical encounter.

A primary strength of our study is its inclusion of two FP clinics that varied in terms of sector, size, and focus on youth-friendly service delivery, thus allowing for examination of how these variables influence implementation. Our use of both inductive and deductive analytic approaches allowed us to relate our findings to existing implementation science knowledge while also leaving room for discovery of contextual factors and/or implementation strategies that did not fit neatly within our chosen analytic frameworks. Our study also had several limitations. First, we only examined two FP clinics, both of which delivered PrEP within the context of a formal research study. Although much of PrEP delivery is being carried out in ways that transition from research to implementation, some of our findings may not apply to, or may overlook factors relevant to, other FP clinics not engaged in research. Second, we did not collect detailed data on strategy dose, thus limiting insight into how intensely strategies must be used to get the observed effect. Third, because we did not collect client experiences of implementation strategies, we cannot comment on whether and how certain strategies affect the acceptability of integrated PrEP-FP delivery. Fourth, since we did not collect data after POWER staff were withdrawn, we cannot comment on the sustainability of these strategies or duration of their effect. And lastly, both of our study’s data sources are subject to response bias, and we did not conduct member checking. Future research should collect client perspectives, specify implementation strategies at study baseline (e.g., actor, action target, and dose), [51] and assess use and effect both qualitatively and quantitatively using additional types of data less subject to response bias, such as direct observations of service delivery.

## Conclusion

With scale up of integrated PrEP-FP services imminent in many parts of SSA, the need to understand which implementation strategies work best under what circumstances is greater than ever. Instead of categorizing potential scale-up sites by the services offered (e.g., “FP clinics”), PrEP implementers should develop a broader range of criteria for assessing sites’ potential for implementation success and link these criteria to implementation strategies that may be needed. This more nuanced approach would allow implementers to better identify “low-hanging fruit” to prioritize for scale-up. It might also enable implementers to better anticipate, and plan for, the heavier “lift” of implementing at facilities that, though less prepared to deliver PrEP to AGYW at baseline, are critical for reaching this population.

## Supplementary Information

**Additional file 1: eTable 1.** Consolidated criteria for reporting qualitative studies (COREQ) checklist. **Appendix A.** Interview guide.

## Data Availability

We are unable to provide access to data, as participants did not give consent to data sharing.
