# Supplementary material for: Implementation strategies for integrating pre-exposure prophylaxis for HIV prevention and family planning services for adolescent girls and young women in Kenya: a qualitative study
Source: BMC Health Serv Res. 2022 Mar 30;22:422. doi: 10.1186/s12913-022-07742-8 (PMC8969252; doi:10.1186/s12913-022-07742-8)
Supplement: Supplementary file 1 — Additional file 1: eTable 1. Consolidated criteria for reporting qualitative studies (COREQ) checklist. Appendix A. Interview guide. [file 12913_2022_7742_MOESM1_ESM.docx]

**Additional File 1 for “Implementation strategies for integrating pre-exposure prophylaxis for HIV prevention and family planning services for adolescent girls and young women in Kenya: a qualitative study”**

**Content:**

**eTable 1**. Consolidated criteria for reporting qualitative studies (COREQ) checklist

**Appendix A**. Interview guide

| **eTable 1. Consolidated criteria for reporting qualitative studies (COREQ) checklist** | | | |
| --- | --- | --- | --- |
| **Domain** | **No. Item** | **Guide Questions/Description** | **Section Where Reported** |
| Research Team and Reflexivity | ***Personal Characteristics*** | | |
|  | 1. Interviewer/facilitator | SDR conducted all interviews. | Methods |
|  | 1. Credentials | SDR has a PhD in Global Health Implementation Science, a Master’s in Public Health, and a BA in Cultural Anthropology. | Methods |
|  | 1. Occupation | At the time these interviews were conducted, SDR was a PhD student and research assistant. | Methods |
|  | 1. Gender | Female | Methods |
|  | 1. Experience and training | SDR has completed doctoral-level courses in qualitative research and first-or co-authored 12 articles on qualitative studies. | Methods |
|  | ***Relationship with Participants*** | | |
|  | 1. Relationship established | Relationships were limited to interviews. Prior to the interview, the participants and interviewer (author SDR) had never met. The interviewer had never before visited the study sites or participated in calls/meetings between interviewees and other POWER staff/co-investigators. | Methods |
|  | 1. Participant knowledge of interviewer | Prior to starting each interview, SDR provided participants with a brief personal introduction, which included that she was not a direct employee of the POWER study but rather a PhD student at the University of Washington investigating participants’ experiences with integrated PrEP-FP delivery for the purposes of understanding what POWER did and what participants felt could be improved upon. During the informed consent process, SDR assured prospective participants of their confidentiality and provided detailed information about steps the study was taking to mitigate the risk of a breach of confidentiality (e.g., removing identifying information from transcripts). | Not included |
|  | 1. Interviewer characteristics | SDR is an American female. | Methods |
| Study Design | ***Theoretical Framework*** | | |
|  | 1. Methodological orientation and theory | Our approach is informed by Corbin and Strauss’s (2015) content analysis and Hsieh and Shannon’s refinement of it. In line with this approach, we collected data primarily through open-ended questions and derived some of our coding categories directly from the text data. | Methods |
|  | ***Participant Selection*** | | |
|  | 1. Sampling | We used purposive sampling to recruit interviewees of different roles and primary employers. | Methods |
|  | 1. Method of approach | The Kenyan study coordinator contacted eligible individuals, described the study’s purpose, and informed them that they would be contacted by an external research assistant about participating in a confidential interview. Prospective participants were informed that the purpose of these interviews was to better understand how PrEP was delivered at each site, the challenges encountered, actions taken in response, and recommendations for other FP clinics that might be interested in adding PrEP to its services. Prospective participants were also informed that the RA conducting the interviews (author SDR) was not a direct employee of the POWER study and that the content of interviews would remain confidential, with any data that could be potentially used to identify participants removed prior to sharing it with other coinvestigators and/or disseminating study results. | Methods |
|  | 1. Sample size | 15 total interviews completed | Results |
|  | 1. Non-participation | All individuals invited for an interview agreed to participate. | Results |
|  | ***Setting*** | | |
|  | 1. Setting of data collection | All interviews were conducted in a private room or via phone call. | Methods |
|  | 1. Presence of non-participants | All interviews were conducted one on one. | Methods |
|  | 1. Description of sample | Our sample included facility staff and POWER staff (employed by the study) involved in PrEP service delivery and/or program implementation. Eligible individuals were age 18 or above and self-reported comfort communicating in English. | Methods, Results, and Table 2 |
|  | ***Data Collection*** | | |
|  | 1. Interview guide | Interviews were conducted using a semi-structured interview guide informed by the CFIR framework. The guide solicited information on participants’ role in PrEP implementation; perceived advantages and disadvantages of the delivery model; strategies used to deliver PrEP; and recommendations for scale-up. | Methods |
|  | 1. Repeat interviews | Each participant was interviewed once at study endline. | Methods |
|  | 1. Audio/visual recording | All interviews were audio-recorded. | Methods |
|  | 1. Field notes | We did not collect field notes for this study. | Not included |
|  | 1. Duration | Interviews typically lasted 85 minutes (interquartile range: 67-90 minutes) | Methods |
|  | 1. Data saturation | We did not conduct interviews to the point of data saturation. | Not included |
|  | 1. Transcripts returned | We did not return transcripts to participants for comment or correction. | Not included |
| Analysis and Findings | ***Data Analysis*** |  |  |
|  | 1. Number of data coders | Two: authors SDR and GB | Methods |
|  | 1. Description of the coding tree | We used a combination of conventional content analysis and directed content analysis, with the latter informed by the ERIC compilation and CFIR framework. | Methods |
|  | 1. Derivation of themes | Our reported themes emerged directly from the text data. | Methods and Results |
|  | 1. Software | Atlas.ti version 8 | Methods |
|  | 1. Participant checking | We did not provide participants with feedback on our findings. | Not included |
|  | ***Reporting*** | | |
|  | 1. Quotations presented | We present participant quotes illustrating our findings. Each quote is accompanied by a participant number. | Results and Table 4 |
|  | 1. Data and findings consistent | Yes | Results |
|  | 1. Clarity of major themes | Our four major themes are identified by subheadings within the Results section. We discuss the relevance of these themes in the Discussion section. | Results and Discussion |
|  | 1. Clarity of minor themes | For Theme 3, we additionally present 4 minor themes related to providers’ perception of their scope of work. We discuss these in detail in the Results and Discussion sections. | Results and Discussion |

**Appendix A. Interview Guide**

**Demographic information**

| What is your gender? | *Male  Female  Other* |
| --- | --- |
| How old are you? | _________ years old |
| What is your position at **[site]**? | *Nurse*  *HTS Counselor*  *Coordinator/manager*  *Other: ___________* |
| In total, how long have you been working as a [position]? | *____months ____ years* |
| How long have you been working as a [position] at **[SITE]**? | *____months ____ years* |
| Do you have any previous experience delivering HIV care or treatment services? *(e.g., counseling, prescribing)* | *Yes  No* |
| How long have you been delivering PrEP at **[SITE]**? | *____months ____ years*  *Not involved in PrEP delivery* |

**Section 1: Background Info**

1. Can you tell me more about the work you do at **[SITE]**?
   1. Main responsibilities *[brief to* ***position participant in the context****, not to obtain detailed explanation of role/responsibilities]*
   2. *[if not already discussed or adequately described]*

Can you tell me more about the work you do with **PrEP**?

1. *[If involved in POWER delivery and/or implementation]*

Before POWER, had you ever delivered PrEP services or been involved in PrEP implementation?

- 1. Were any of those experiences focused on PrEP **for** **young women**?

**Section 2: PrEP Delivery Model – What works well, for whom, why?**

*[For participants involved in delivering PrEP services (e.g., counselors, nurses) and/or monitoring/coordinating PrEP service delivery in POWER (e.g., coordinators, PIs]*

1. Based on your experience, what would you say are some of the **advantages** of delivering PrEP through **[the FP clinic of a hospital]**? Why do you think that?
   - 1. Probe about **how site type may facilitate access to young women**.

***Does a hospital attract certain populations? Why would some women choose to go to a FP clinic within a hospital, rather than a stand-alone FP clinic? Who self-select to go to a hospital FP clinic?***

- 1. What are some of the **disadvantages**? Why do you think that?
  2. In general, how well do **[FP clinics]** like **[SITE]** understand young women’s needs? Why do you think that?

1. ***I’d like to talk about some of the things that have worked well here at [SITE]. Let’s start with demand creation.*** What are some of the things that **[SITE]** **does well** to **reach young women** who may benefit from PrEP? Why do you think that?
   - 1. Probe about things that happen at the site (e.g., health talks, showing video, in reaches) and in the community (e.g., flier distribution, events)
   1. Have these things worked better for some young women than others? With whom and why do you think that?
      1. ***When s/he says what works better for some and others, is that based on what you have seen or what you think?***
   2. Are there any **unexpected results or challenges** to [these things]?
   3. What **hasn’t** worked as well? Why do you think that?
   4. **Without POWER**, how easy or difficult would it be for **[SITE]** to implement [these things] in the community? Why do you think that?

*[Note: If participant talks about challenges with scale-up, keep these in mind for later questions]*

1. What are some of the things that **[SITE]** does well to help young women **decide to start** PrEP? Why do you think that?
2. What **messages or materials** have worked well? Why do you think that?
   - 1. **[Probe if needed]** Have you noticed that messages are framed in any particular way that help women decide to start PrEP?
3. Have [these things] worked better for some young women than others? With whom and why do you think that?
4. Are there any **unexpected results or challenges** to [these things]?
5. What **hasn’t** worked as well? Why do you think that?
   - 1. **[Probe if needed]** Besides messages, is there anything else [SITE] has tried to do to support decision-making that hasn’t worked?
6. What are some of the things that **[SITE]** does well to help young women **adhere to and persist on PrEP**? Why do you think that?

**[Probe as needed:]** Why do you think these things work? Is this based on what you have seen in terms of outcomes when these messages are used? Based on what you have heard from clients? What evidence are these opinions based on? What are the underlying mechanisms that make these work for young women?

1. What **on-going help** do young women taking PrEP need and what **strategies** have helped over time?
2. Why do you think **drop-off** occurs at **[SITE]** currently? Why do you think that?
3. What messages or materials have worked well? Why do you think that?
4. Have [these things] worked **better for some** young women than others? With whom and why do you think that?
5. Are there any **unexpected results or challenges** to [these things]?
6. What **hasn’t** worked as well? Why do you think that?

*[For Questions 7-8, if participant does not routinely deliver services (e.g., site coordinators, PIs), ask about ‘PrEP services’ not specified based on their position/services they deliver]*

1. Based on your experience as a [position], in what ways are [the PrEP-related services your position specifically does, e.g., counseling, prescribing, dispensing] at **[SITE]** delivered to **better meet the needs** of young women?
2. Probe about **site-specific strategies** e.g., service bundling, in-room phlebotomy/dispensing, support groups, differentiated follow-up/refill

**[Probe as needed]** What services are bundled with PrEP service delivery?

1. **How well** do [these things] work? What are the **challenges** of [these things]?
2. Was this how [the services] were always delivered at **[SITE]** or were these **changes** you or your colleagues **made to better meet young women’s needs**?
3. What aspects of [services] **still do not work well** for young women or are still burdensome for young women? Why do you think that is?
4. To what extent did you feel that [services] at **[SITE]** could be adapted or refined to better fit young women’s needs?
5. Based on your experience as a [position], in what ways are [the PrEP-related services your position specifically does, e.g., counseling, prescribing, dispensing] at **[SITE]** delivered to better meet **the needs of the site or staff in positions like yours**?
   - 1. Probe about **site-specific strategies** e.g., integrated service delivery, patient flow, task-shifting
6. How well do those work? What are the challenges of these? How challenging would these be to carry out **without dedicated POWER staff**?
7. Imagine there were no research procedures. If you think only about how [the services your position specifically does] are carried out, what feels **out of place, impractical, or burdensome** for **[SITE]**? Why do you think that is?
8. What might feel fine for you now, but may be impractical or burdensome for to do **once you [if POWER staff]/POWER is gone**?
9. To what extent did you feel that [the services your position specifically does, e.g., counseling, prescribing, dispensing] at **[SITE]** could be **changed or improved** to better fit the site or [positions]?

**Section 3: Implementation of PrEP Service Delivery Model**

*[For participants involved PrEP service delivery/implementation in POWER]*

1. How familiar are you with how PrEP service delivery for young women was **introduced** at **[SITE]**? *[If familiar, ask the following probes, if not familiar skip to next question].*
   1. How were **leaders or staff involved** in planning for PrEP implementation? How did the leaders or staff at **[SITE]** feel about PrEP for young women? About PrEP being implemented here?
   2. How important was implementing PrEP **compared to other priorities** at **[SITE]**?
   3. What **resources** did the site make available for PrEP implementation?
2. I would like to learn more about your opinions on how PrEP service delivery is implemented at **[SITE]**.
   1. Who has helped lead implementation?
   2. What have they done to make the process **easier**?
   3. What are some of the **challenges**?
   4. Are there any specific people that help **motivate** others to deliver PrEP? What do they do?
   5. Do you think **the right people** are involved in PrEP implementation? Why? Who else should be involved either within or outside of **[SITE]**?
   6. In your opinion, how well do you think **[SITE]** was **linked with other sites** locally, nationally, or internationally engaged in delivering PrEP?
   7. What, if any, **external factors**—such as changes in guidelines or policies—have influenced PrEP service delivery and/or implementation at **[SITE]**?
   8. Over the time you were involved in delivering PrEP, did you receive **any feedback** about how delivery was going? If so, how do you or the team use that information?
3. **Before you started**, in general, what did you think about PrEP for young women?
4. **What did you know** about PrEP before you started?
5. Why did you want to or not want to deliver PrEP services?
6. What were your **concerns**?
7. What made it **easier or harder** **to start** delivering PrEP services to young women?
8. How **confident** were you that PrEP could prevent HIV infection?
9. How did you feel about PrEP **compared** to other HIV prevention services?
10. Now that you have been delivering PrEP services to young women for [time period], how do you feel about it? Why do you feel that way?
11. What makes it **easy or difficult** to deliver PrEP services?
12. How **confident** are you that you can provide **quality PrEP services** to young women? Why? What would make you feel more confident?
13. If you have questions about PrEP or about PrEP services, where or who do you go to for information? Is that helpful to you?
14. What types of **on-going support** do you feel you need to successfully deliver PrEP services?
15. Is there anything about delivering PrEP that has **surprised you** (e.g. unexpected challenges, enthusiasm, strategies to overcome challenges, etc.)?

**Section 4: Considerations for Scaling the PrEP Service Delivery Model**

*[For participants involved PrEP service delivery/implementation in POWER]*

We have talked a lot about how PrEP services for young women are delivered at **[SITE]** and what works and what doesn’t work. Imagine that **[Kenya]** wanted to scale PrEP service delivery to 20 other **[FP clinics]** as part of their national PrEP programme. By this I mean to say scale-up would be “real-world” **without the additional staffing of POWER** but potentially still using the educational/counseling tools or support strategies developed in POWER.

1. What about PrEP service delivery for young women here at **[SITE]** do you think is **essential** and would have to be a part of PrEP service delivery for young women at these 20 other **[FP clinics]** in order for it to work? Why do you think that?
   - 1. Probe specifically about topics discussed in Theme 2 (things done to reach AGYW at risk, to support YW’s decision to take PrEP, support YW to adhere to and persist on PrEP, to support YW’s needs, to support clinic/healthcare provider’s needs).
     2. *[If not discussed already]* Probe specifically about **service bundling** (e.g., **what other services are helpful/needed by young women?** (e.g., STI testing and treatment, FP, mental health services, social support)
2. How easy or hard would it be to include these things? Why?
3. What about PrEP service delivery for young women at **[SITE]** could be **modified or changed** in these 20 **[FP clinics]** to improve its success?
   - 1. Probe specifically about topics discussed in Theme 2 (things done to reach AGYW at risk, to support YW’s decision to take PrEP, support YW to adhere to and persist on PrEP, to support YW’s needs, to support clinic/healthcare provider’s needs).
     2. *[If not discussed already]* Probe specifically about how delivery could be modified or changed—**How might delivery be modified or changed** **to reduce the drop off after PrEP initiation? What changes do you think could help?** (e.g., give young women a follow-up appt rather than same day PrEP start, group sessions before starting PrEP, more contact in the first couple of weeks)
   1. How easy or hard would it be to make these changes? Why?
4. What would be the **major challenges to scaling** PrEP services in other **[FP clinics]**?
5. What could be done to minimize these challenges?
6. Is there anything else you think someone who is scaling up PrEP service delivery to other **[FP clinics]** should think about?

**Section 5: Broader Landscape of PrEP Service Delivery to Young Women**

[*Cover the following questions with key informants with broader view of landscape of PrEP delivery to young women, i.e., site PIs, site coordinators, other KIs as determined with sites].*

*[For KIs who will also cover Themes 2-4 but are not involved in day-to-day PrEP service delivery and/or implementation in POWER (e.g., site PIs), Theme 5 may be covered before Themes 2-4].*

1. When POWER started, there were very few existing efforts at trying to deliver PrEP to young women, whereas now, there are many such projects.

**How familiar are you with other efforts in Kenya or elsewhere to deliver PrEP to young women?**

*If not familiar, skip to next question]*

1. How are the challenges these projects face **similar or different** from the ones we’ve seen in POWER?
2. What do you think are the **advantages** to delivering a new intervention (like PrEP) in a more controlled study, such as POWER, as compared to a larger roll-out in routine clinic settings? What are some of the **disadvantages**?
3. What do you think are the **three biggest barriers to population-level success or effectiveness** of PrEP delivery to young women?
4. Are you aware of any PrEP delivery model or project that has overcome (or made strides to overcome) those barriers? If so, what did they do?
   - 1. *[Only if yes to above]* Would the strategy described above be feasible to implement in routine clinic settings? Why/why not?
5. *[If not already discussed above]* We have heard that a major challenge to making public health impact with PrEP for young women is **creating demand** for PrEP.

**Do you have any suggestions/thoughts about proven or promising strategies to create demand for PrEP among young women?**

1. *[If not already discussed above]* We have also heard that another major challenge to making a public health impact with PrEP for young women is **lack of persistence**.
   1. What do you think **optimal persistence** is for young women?
   2. How do you interpret the **large drop-off after initiation** that has been observed across many PrEP projects among AGYW?
   3. Do you have any suggestions/thoughts about proven or promising strategies to ensure young women at risk continue to take PrEP?
2. We’ve heard from some other PrEP delivery projects—ones where there is supplemental staff provided to the clinic to help deliver PrEP—that one of the biggest challenges for continuing the program is **concerns about patient load and the corresponding workload**.
3. With workload and congested facilities in mind, what would you consider the **minimum package of services** that must be delivered along with PrEP?
   - 1. **[Probe as needed]** **Why** should these services be included in the minimum package? Is including these services in the minimum package feasible/acceptable in the ‘real-world’?
     2. **[If STI services mentioned]** Do you mean STI diagnostic services or screening?
4. If you were to be able to add one additional service (while retaining engagement of HCP), what would it be?
5. Probe about STI screening, syndromic vs lab,
6. **[Probe as needed]** **Why** would you include this service? Would it be **feasible and acceptable** to include this additional service in the minimum package in public health facilities?
7. *[Only if not asked under Theme 3 above]*

Is there anything about delivering PrEP to young women that has **surprised you** (e.g. unexpected challenges, enthusiasm, strategies to overcome challenges, etc.)?

**Section 6: Wrap-up**

1. How would you complete the following sentence? *[if not already answered above]*
2. PrEP will never be delivered to a large enough quantity of young women for a public health impact unless projects can figure out how to______________________.
3. Outside of how PrEP service delivery is organized in the clinic, it is essential that _________________be in place in the broader environment if PrEP delivery to young women is to succeed.
4. Is there anything else we should know about PrEP service delivery to young women or questions we should have asked?
